# Evaluating the Applicability of Phi Coefficient in Indicating Habitat Preferences of Forest Soil Fauna Based on a Single Field Study in Subtropical China

**DOI:** 10.1371/journal.pone.0150380

**Published:** 2016-03-01

**Authors:** Yang Cui, Silong Wang, Shaokui Yan

**Affiliations:** 1 Institute of Applied Ecology, Chinese Academy of Sciences, Shenyang, China; 2 Huitong National Research Station of Forest Ecosystem, Huitong, China; 3 University of Chinese Academy of Sciences, Beijing, China; Chinese Academy of Forestry, CHINA

## Abstract

Phi coefficient directly depends on the frequencies of occurrence of organisms and has been widely used in vegetation ecology to analyse the associations of organisms with site groups, providing a characterization of ecological preference, but its application in soil ecology remains rare. Based on a single field experiment, this study assessed the applicability of phi coefficient in indicating the habitat preferences of soil fauna, through comparing phi coefficient-induced results with those of ordination methods in charactering soil fauna-habitat(factors) relationships. Eight different habitats of soil fauna were implemented by reciprocal transfer of defaunated soil cores between two types of subtropical forests. Canonical correlation analysis (CCorA) showed that ecological patterns of fauna-habitat relationships and inter-fauna taxa relationships expressed, respectively, by phi coefficients and predicted abundances calculated from partial redundancy analysis (RDA), were extremely similar, and a highly significant relationship between the two datasets was observed (Pillai's trace statistic = 1.998, *P* = 0.007). In addition, highly positive correlations between phi coefficients and predicted abundances for Acari, Collembola, Nematode and Hemiptera were observed using linear regression analysis. Quantitative relationships between habitat preferences and soil chemical variables were also obtained by linear regression, which were analogous to the results displayed in a partial RDA biplot. Our results suggest that phi coefficient could be applicable on a local scale in evaluating habitat preferences of soil fauna at coarse taxonomic levels, and that the phi coefficient-induced information, such as ecological preferences and the associated quantitative relationships with habitat factors, will be largely complementary to the results of ordination methods. The application of phi coefficient in soil ecology may extend our knowledge about habitat preferences and distribution-abundance relationships, which will benefit the understanding of biodistributions and variations in community compositions in the soil. Similar studies in other places and scales apart from our local site will be need for further evaluation of phi coefficient.

## Introduction

Habitat preference is an important factor that influences the distribution of organisms [1] and thus is a vital ecological process that explains the composition of biological communities [2]. As a result, knowledge about habitat preferences, such as relationships with habitat factors, is necessary to understand the distribution of organisms and variations in community compositions.

In a terrestrial ecosystem, soil fauna play important roles in soil nutrient processes and the formation and preservation of soil physical structure [3–7], and thus, their relationships with habitat factors have drawn considerable attention [8–11]. Meanwhile, soil fauna assemblages, such as the community of Nematode or Collembola, have been used as indicators of soil conditions and ecosystem status [12–14] due to their sensitivities and narrow requirements for habitat conditions. Based on coarse levels of taxonomic resolution, an abundance-based fauna index for assessing soil quality has been proposed [15]. While various qualitative methods, such as canonical ordination, have been widely used to study the correlations between soil fauna and habitat (factors), the relationships between habitat preferences of soil fauna and habitat factors have not been well studied. Moreover, studies have not investigated these relationships quantitatively, as the results on degrees of habitat preferences in previous studies were mostly qualitative or semi-quantitative [16–18]. Quantifying the degree of habitat preferences and the associated relationships with habitat factors could be beneficial for accurately determining the factors underlying the distributions of soil fauna, and would present a lot of interest for conservation biology, forest management and other applications.

In vegetation ecology, phi coefficient of association has been widely used to evaluate the strength of association between organism assemblages and site groups, indicating the degree of preference for the target site group compared to other site groups [19–21]. However, in soil ecology, application of this correlation index to analyse habitat preferences of soil fauna has not been reported. The following properties of phi coefficient are useful for indicating and comparing the degree of preferences for site groups [19, 21]: (1) it is independent of the number of observations and modestly affected by the relative size of the target site group; (2) the calculations of phi coefficients with a site group are independent among species, so the pattern for a given species does not influence the strength of association between another species and the site group; and (3) the value of a phi coefficient is bounded and can be negative, indicating that a species may tend to “avoid” particular environmental conditions. As a result, phi coefficient might be suitable for quantifying the strength of habitat preferences of soil fauna, provided the number of site groups is adequate. And the relationships between habitat preferences and habitat factors might be further obtained by using statistical methods such as correlation analysis and regression analysis. However, the relationships of soil fauna with habitat (factors) are usually blurred by the heterogeneity of sampling locations. Consequently, related studies have to extract variation information originating from sampling locations before acquiring the desired results [11]. Commonly, sampling locations are not only permanent habitats but also the provenance of soil fauna. Accordingly, it can be an appropriate approach to incubate different forest soils in the same provenance to compare the degree of habitat preferences.

The results of constraint ordination can reflect abundance-habitat relationships and abundance-habitat factor relationships when phi coefficient-induced results indicate the strength of habitat preferences and the associated relationships with habitat factors. We therefore compared the phi coefficient-induced results with those generated from the constraint ordination method to evaluate the local-scale applicability of phi coefficient in quantifying the degree of habitat preferences of soil fauna. A single field experiment involving different habitats for soil fauna formed by reciprocal transfers of defaunated soil cores between two types of forest was implemented in subtropical China, based on which the phi coefficient was evaluated.

## Materials and Methods

### Study site and experimental design

Our study was permitted by Huitong National Research Station of Forest Ecosystems (HNRSFE) in China in their experimental forests (26° 50’N, 109° 36’E). No specific permissions about our study were required by HNRSFE, because that this study only involved soil invertebrates, and no endangered or protected species inhabited in their experimental forests. The topography of this area is characterized by low mountains (elevation 300–500 m), and the main forest types are secondary evergreen broadleaf forests, *Cunninghamia lanceolata* plantations and *Pinus massoniana* plantations. The forest soils are mainly reddish yellow soils (Ultisols) and yellow soils (Inceptisols).The mean annual precipitation is 1200–1400 mm, and the mean annual temperature is 16.5°C with an annual mean air humidity of 80%.

**Fig 1:**
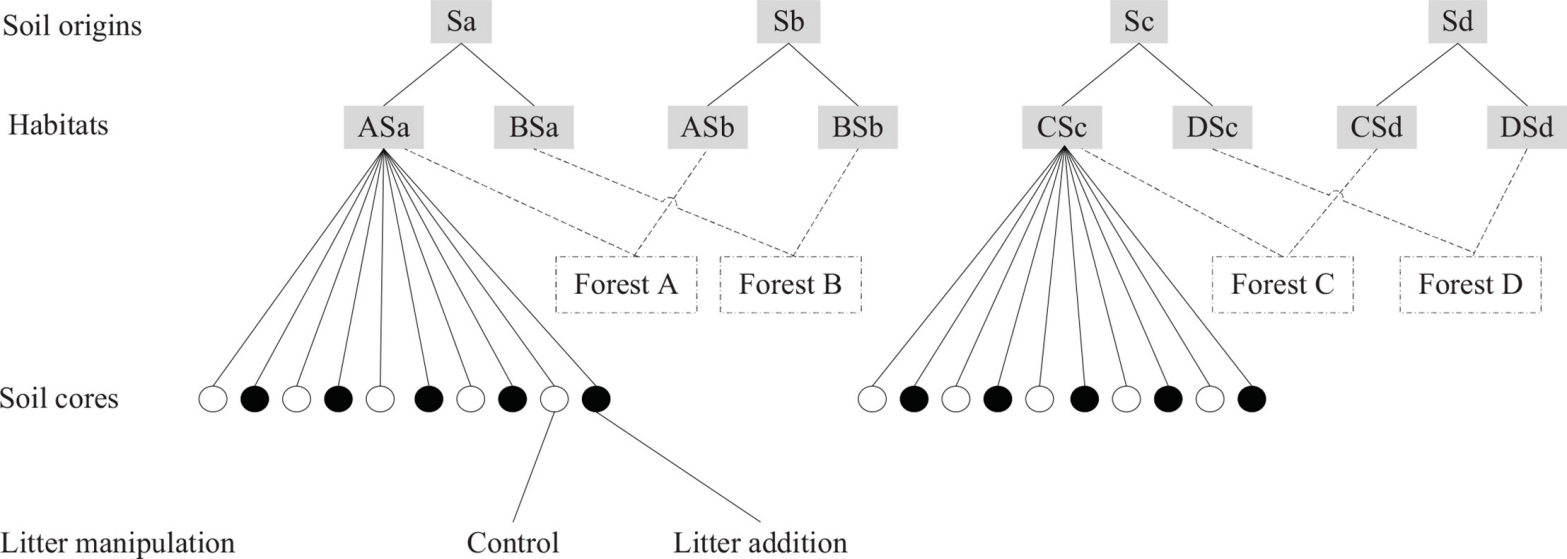
Experimental design sketch for this study. Sa, soils originating from forest A; Sb, soils originating from forest B; Sc, soils originating from forest C; Sd, soils originating from forest D.

Eight different habitats for soil fauna were created by reciprocal transfers of defaunated soil between two types of forest involving two *C*. *lanceolata* plantations (labelled as B and D) established in 1983 and two secondary evergreen broadleaf forests (labelled as A and C) naturally regenerated from clear-cutting of *C*. *lanceolata* in 1996. The dominant tree species in forests A and C were *Castanopsis fargesii*, *Machilus pauhoi*, *Alniphyllum fortune*, and *Cyclobalanopsis glauca*. In each forest, a rectangular area of approximately 30 m × 12 m was demarcated. Because of the irregular topography, six to eight parallel transects 1.5 m apart were established, and along each transect, 10 to 25 points were located 1 m apart. In total, 160 spots were marked and numbered. Then, 25 spots were randomly selected for soil sampling. On each selected spot, the 0–17 cm of topsoil was collected using a sampler 13 cm in diameter. The collected soil was defaunated through high-pressure steam sterilization and then air-dried and mixed. Meanwhile, in each forest, 20 of the 25 pits dug during sampling were randomly selected for the subsequent incubation of defaunated soil. Among the selected 20 pits in forest A, 10 pits were randomly selected and filled with defaunated soil originating from forest A, and the other 10 pits were filled with defaunated soil originating from forest B, and vice versa for the selected 20 pits in forest B. Similarly, another reciprocal soil transfer was conducted between forests C and D. In this way, eight different habitats, each containing 10 soil cores, were framed for soil fauna and coded as ASa, ASb, BSa, BSb, CSc, CSd, DSc, and DSd with labels Sa, Sb, Sc, and Sd indicating soils originating from forests A, B, C, and D, respectively (Fig 1). In forests A and C, half of the transferred and un-transferred soil cores were covered with 10 g of dry weight fresh leaf litter of *C*. *fargesii* while the other half was covered not with litter but with plastic threads to simulate the physical effect of a litter covering. Similar treatment of litter additions was carried out in forests B and D using leaf litter of *C*. *lanceolata*. The effects of litter on soil fauna were not the focus of this study, but litter treatment was involved in the statistical tests to obtain reliable results. This field experiment was established from 25 April to 18 May 2011. The soil cores incubated in the fields were harvested 437 days later, and then fauna extraction and microbiological and chemical measurements were initiated.

### Soil sampling

For each soil core, soil sampling for fauna extraction was carried out using a metallic tube 5 cm in diameter from three soil depths (0–5 cm, 5–10 cm and 10–15 cm) twice, each corresponding to two different extraction apparatuses. The remaining soil after fauna sampling in each soil core was 2 mm deep, which was sieved and mixed sufficiently. Part of the 2-mm sieved soil was stored at 4°C for determination of soil microbial biomass C (MBC, mg kg^-1^), basal respiration (BR, mg CO_2_ kg^-1^h^-1^) and dissolved organic C (DOC, mg kg^-1^) and inorganic N (NH_4_^+^ / NO_3_^-^, mg kg^-1^). The rest was air-dried and ground more finely for determination of soil pH, TOC (g kg^-1^), total nitrogen (N, g kg^-1^), and total phosphorus (P, g kg^-1^).

### Laboratory analysis

Two different fauna extraction apparatuses were used. One apparatus was a Tullgren dry funnel, which mainly extracts microarthropods such as Acari and Collembola [22]. The other was a Baermann funnel, which mainly extracts hydrobionts such as nematodes and Enchytraeids [23]. Fauna samples were preserved in 70% alcohol for identification and counting. The abundance of each taxon was determined in terms of density (ind.m^-2^). MBC was determined by the chloroform fumigation–extraction method [24], and DOC was determined by measuring the content of organic carbon in the extract of un-fumigated soil during measurement of MBC. Inorganic N was extracted with a 2 *M* KCl extracting water solution, and then the NH_4_^+^ and NO_3_^-^ in the extract were measured colorimetrically [25]. TOC was determined by a potassium dichromate external heating method [26], the pH was determined by a potentiometric method in water (1: 2.5 soil: water), N was determined by the semi-micro Kjeldahl method [27], BR was determined by the methods described by Wang and Wang [28], and P was determined by colorimetrically using the ammonium molybdate stannus chloride method [29].

### Data analysis

In total, 18 fauna taxa were identified and Kruskal-Wallis test was used for comparing the mean abundances and relative abundances of fauna taxa between habitats (S1 Table). Taxa encountered in only one sample, which included Lepidoptera larvae, Harpacticoida, Coleoptera, Homoptera, Geophilomorpha, were removed from the fauna abundance dataset. Consequently, the remaining 13 taxa were involved in the following analyses. A detrended correspondence analysis (DCA) of fauna abundance was performed to measure the length of the ecological gradients. The length of the first DCA axis was 2.73, which indicated a linear response along the axis [30], and a partial redundancy analysis (RDA) [31] was conducted to explore the relationships between soil fauna (response variables) and soil chemical properties (explanatory variables) with litter treatment and habitats as covariables. All of the soil chemical variables were standardized and the fauna abundance data were Hellinger-transformed prior to the partial RDA. The Hellinger transformation allowed Euclidean distances to be used in the partial RDA while giving low weights to rare species [32]. The linear dependencies between explanatory variables in the partial RDA model were explored by computing the variables’ variance inflation factors (VIF) [33]. No variables surpassed a VIF value of 5, indicating that there was no severe collinearity in the model [31]. Meanwhile, permutation tests (999 permutations) were carried out for the partial RDA model.

In the RDA biplot, the relationship between the centroid of a site group and a fauna taxon was found by projecting the centroid at a right angle on the species arrow, which could approximate the average abundance of the taxon within the group [30, 31]. As a result, the ordering of habitats on the projection values predicted the ordering of the habitats regarding abundances. The formula for calculating the projection values is shown below (*Eq* 1*)*. Here, negative projection values for a site group predicted that the abundance of the taxon within the group was lower than the mean levels represented by the biplot origin.

$$Projection\;value\;of\;centroid=\frac{C_{rda1}\times S_{rda1}+C_{rda2}\times S_{rda2}}{\sqrt{S_{rda1}^{2}+S_{rda2}^{2}}}$$ Eq. 1

*C*_*rda1*_: the centroid scores of dummy variables along the 1^st^ RDA axis;

*C*_*rda2*_: the centroid scores of dummy variables along the 2^nd^ RDA axis;

*S*_*rda1*_: the species scores along the 1^st^ RDA axis;

*S*_*rda2*_: the species scores along the 2^nd^ RDA axis.

Conversely, the presence/absence dataset transformed from fauna abundances was used for calculation of phi coefficients [19, 21] to determine the strength of habitat preferences of soil fauna with the multipatt function of the indicspecies package [34] in R. The formulae for phi coefficient are shown below (Table 1: *Eq*. *2* and *3*). *Eq*. *3* is a modification of *Eq*. *2* when site groups have unequal sizes [20], which allows comparisons between values corresponding to site groups of different relative sizes. If all habitats originally had the same size, then $\gamma_{\Phi }=\gamma_{\Phi }^{\text{g}}$ [21]. As a result, in the present study, $\gamma_{\Phi }^{\text{g}}$ was used for calculation of phi coefficients even though the eight habitats had the same sizes (each was 10 soil cores).

**Table 1:** Phi coefficient of association between a species and a group of sites.

	Formulae	Groups size equalized
***Eq*. *2***	$\gamma_{\Phi }=\frac{N\times n_{p}-n\times N_{p}}{\sqrt{\left(N\times n-n^{2}\right)\times \left(N\times N_{p}-N_{p}^{2}\right)}}$	non-equalized
***Eq*. *3***	$\gamma_{\Phi }^{\text{g}}=\frac{N\times n_{p}^{\text{g}}-n^{\text{g}}\times N_{p}^{\text{g}}}{\sqrt{\left(N\times n^{\text{g}}-{n^{\text{g}}}^{2}\right)\times \left(N\times N_{p}^{\text{g}}-{N_{p}^{\text{g}}}^{2}\right)}}$	equalized

*N*, total number of sites; *N*_*p*_, number of sites belonging to the target site group; *n*, number of occurrences of the species among all sites; *n*_*p*_, number of occurrences of the species within the target site group. In addition to the quantities defined in *Eq*. *2*, the following symbols are used: *K*, number of site groups; *N*_*k*_, number of sites belonging to the *kth* site group; *n*_*k*_, number of occurrences of the species in the *kth* site group; $N_{p}^{g}=N/K;\;n_{p}^{g}=N_{p}^{g}(n_{p}/N_{p})$; $n^{g}=N_{p}^{g}\times \sum_{K=1}^{K}\left(n_{K}/N_{K}\right)$.

After calculating the phi coefficients, the general correlation involving Pillai's trace statistic between the phi coefficients matrix and predicted abundances matrix was tested using canonical correlation analysis (CCorA) based on standardized data. Only six abundant fauna taxa, i.e., Acari, Collembola, Hymenoptera, Nematode, Enchytraeid, and Dipteralarvae, were included in the CCorA because this method required the total number of variables in the two matrices to be smaller than (n − 1). In the present study, n represented the number of habitats and was equal to 8. The abundant fauna taxa were shown in a partial RDA biplot and by Kruskal-Wallis tests of abundance and relative abundances (S1 Table). For all 13 fauna taxa associated with the partial RDA, the quantitative relationships between phi coefficients and predicted abundances were evaluated using linear regression analyses. Additionally, the phi coefficients for fauna taxa were regressed on soil chemical variables to determine their relationships. All of the aforementioned analyses were conducted in R 3.1.1 [35] and the multivariate analyses were carried out using the Vegan package [36].

## Results

### The relationships between soil fauna and soil chemical variables

**Fig 2:**
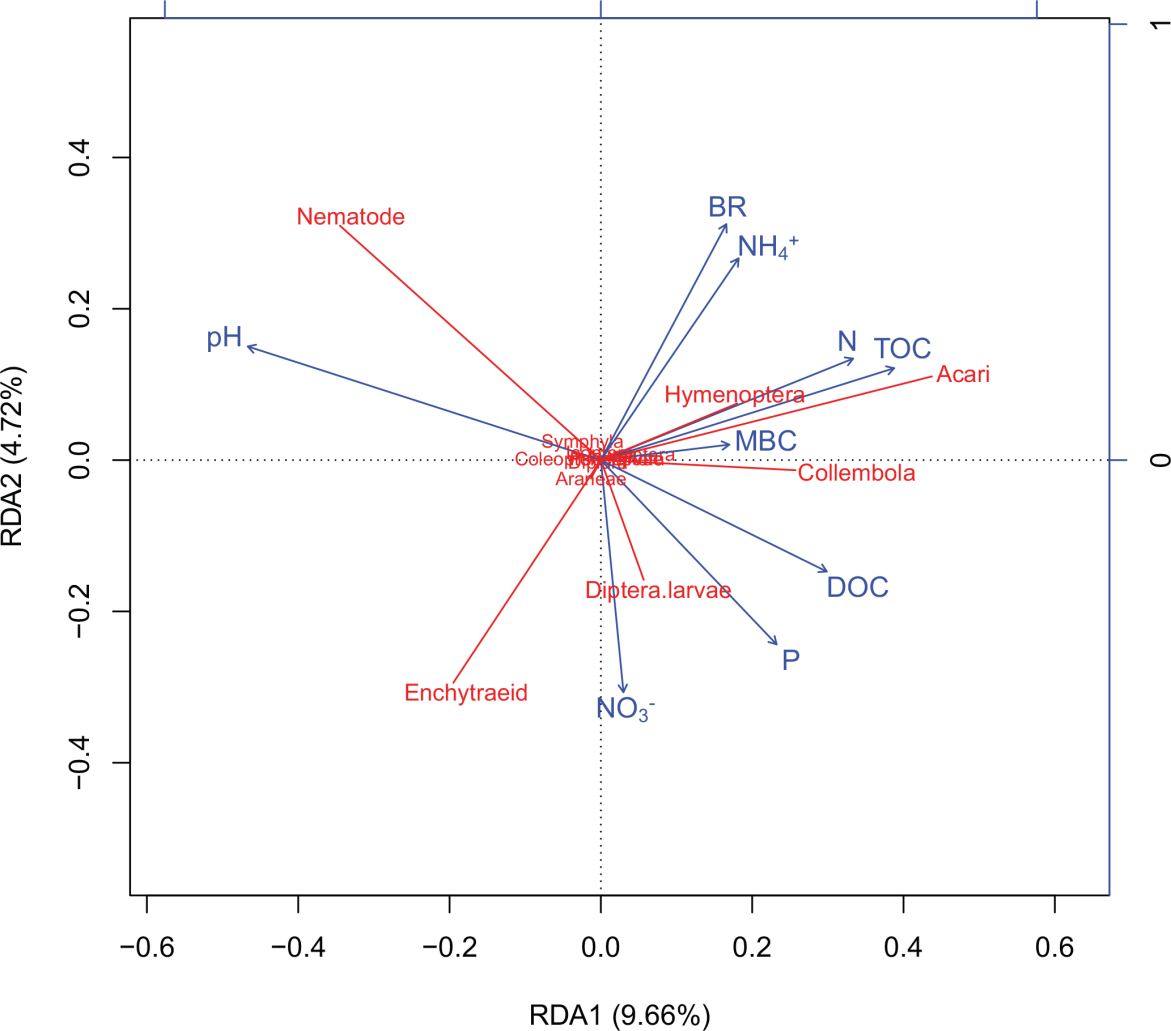
The partial RDA diagram showing the relationships between soil fauna and soil chemical variables.

The global permutation test showed the partial RDA model was significant (F-ratio = 1.54; *P =* 0.04). In total, the constrained variables accounted for 16.5% of the variation in the soil fauna community. The first canonical axis alone explained 9.66% of the variance (F-ratio = 8.00; *P =* 0.0002; Fig 2), and the second axis explained 4.72% (F-ratio = 3.91; *P =* 0.009; Fig 2). Jointly, the first two axes explained 14.4% of the variance. The partial RDA biplot showed that Hemiptera, Symphyla, Coleoptera larvae, Araneae, Diplura, Psocoptera, and Isoptera were located aggregately around the middle of the ordination plot with shorter arrows, showing their lower predicted abundances and less importance to the first two axes, while the other taxa, such as Hymenoptera, Acari, Collembola, Enchytraeid, Nematode, Diptera larvae with longer arrows were more abundant and important (Fig 2).

The positive direction of the first axis was characterized by an increasing gradient of TOC, DOC and N and a decreasing gradient of pH when the second axis was characterized by an increasing gradient of BR, NH_4_^+^ and a decreasing gradient of NO_3_^-^ and P (Fig 2). Along the first axis, fauna taxa Acari, Collembola, and Hymenoptera were positively correlated with N, TOC, and DOC and negatively correlated with pH, while Nematode was positively correlated with pH and negatively correlated with DOC. Along the second axis, Enchytraeid had a highly negative correlation with BR and NH_4_^+^ and a positive correlation with NO_3_^-^, and Nematode was negatively correlated with P and NO_3_^-^. In addition, along the second axis, Diptera larvae was positively correlated with NO_3_^-^ and P and was negatively correlated with BR and NH_4_^+^.

**Fig 3:**
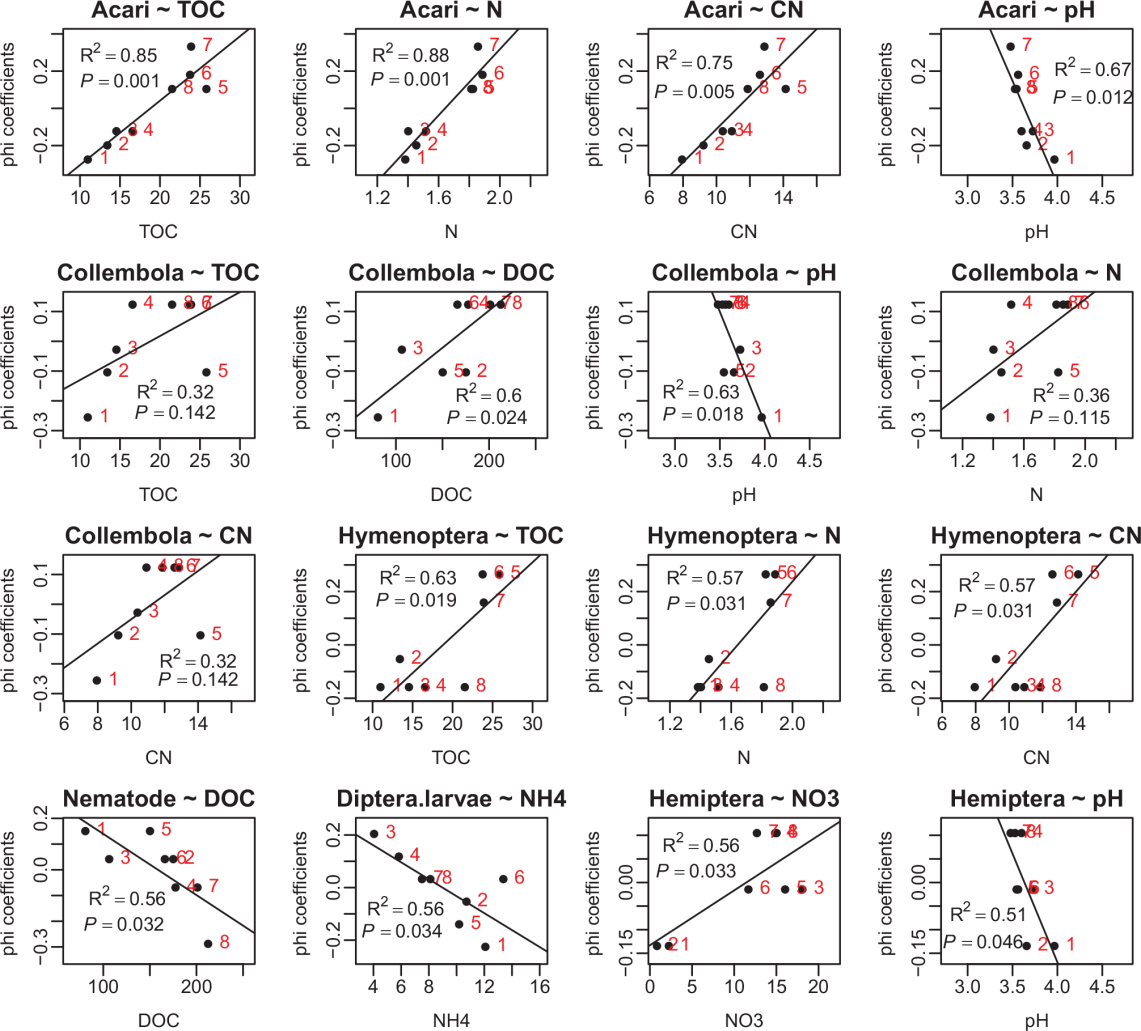
Relationships between phi coefficients and soil chemical variables. Codes for habitats: 1 –ASa; 2 –ASb; 3 –BSa; 4 –BSb; 5 –CSc; 6 –CSd; 7 –DSc; 8 –DSd.

Additionally, quantitative relationships between phi coefficients and soil chemical variables were determined by linear regression analyses (Fig 3). The phi coefficient for Acari was positively correlated with TOC (R^2^ = 0.85, *P* = 0.001), N (R^2^ = 0.88, *P* = 0.001) and C/N (R^2^ = 0.75, *P* = 0.005) and was negatively correlated with pH (R^2^ = 0.67, *P* = 0.012). The phi coefficient for Collembola was positively correlated with DOC (R^2^ = 0.60, *P* = 0.024) and negatively correlated with pH (R^2^ = 0.63, *P* = 0.018). The phi coefficient for Hymenoptera was positively correlated with TOC (R^2^ = 0.63, *P* = 0.019), N (R^2^ = 0.57, *P* = 0.031) and C/N (R^2^ = 0.57, *P* = 0.031). The phi coefficient for Nematode was negatively correlated with DOC (R^2^ = 0.56, *P* = 0.032). The phi coefficient for Dipteral larvae was negatively correlated with NH_4_^+^ (R^2^ = 0.56, *P* = 0.032). The phi coefficient for Hemiptera was positively correlated with NO_3_^-^ (R^2^ = 0.56, *P* = 0.033) and negatively correlated with pH (R^2^ = 0.51, *P* = 0.046).

### The relationships of phi coefficients with predicted abundances of soil fauna

**Fig 4:**
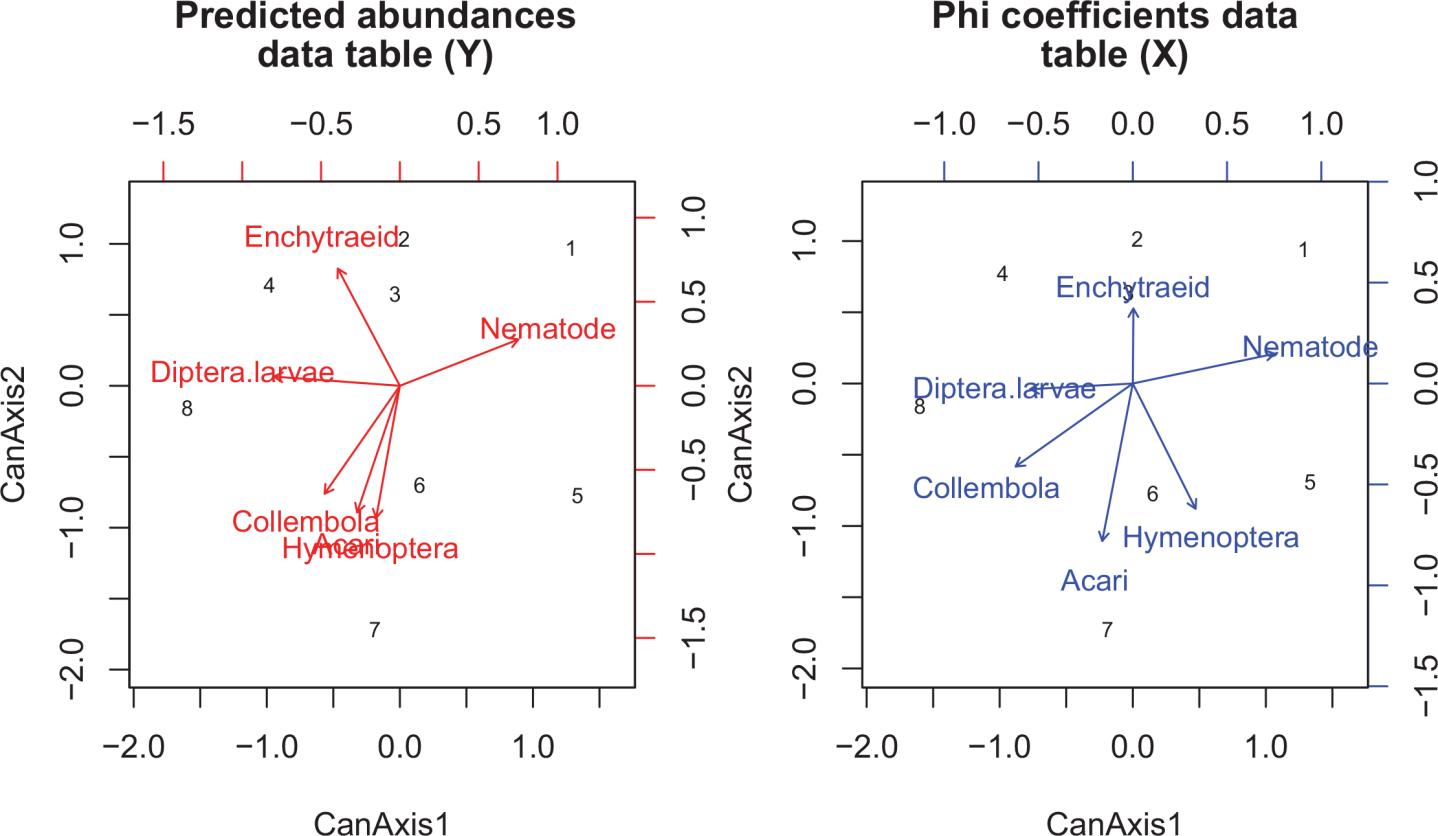
Biplots of a canonical correlation analysis (CCorA) of the predicted abundances (*left*) and phi coefficients (*right*) of abundant soil fauna. Codes for habitats: 1 –ASa; 2 –ASb; 3 –BSa; 4 –BSb; 5 –CSc; 6 –CSd; 7 –DSc; 8 –DSd.

The results of CCorA showed a highly significant relationship between the phi coefficients matrix and predicted abundances matrix, and similar structures (Fig 4). The canonical correlations were high on the first two axes (1 for canonical axis 1, 0.999 for canonical axis 2) and Pillai's trace statistic was 1.998 (*P* = 0.007). In Fig 4, the left-hand biplot showed the standardized predicted abundances of soil fauna and habitats projected in their space. The right-hand biplot showed the standardized phi coefficients of soil fauna and habitats projected in their space. The two spaces were strongly “aligned” with respect to one another, and the canonical axes showed the same trends expressed as similar relationships among soil fauna taxa and the extremely related positions of habitats in the two biplots. The pair of biplots expressed the fact that for Acari, Collembola, Nematode and Diptera larvae, the predicted abundances were highly positively correlated with phi coefficients, whereas for Hymenoptera and Enchytraeid, the relationships were weakly positive.

**Fig 5:**
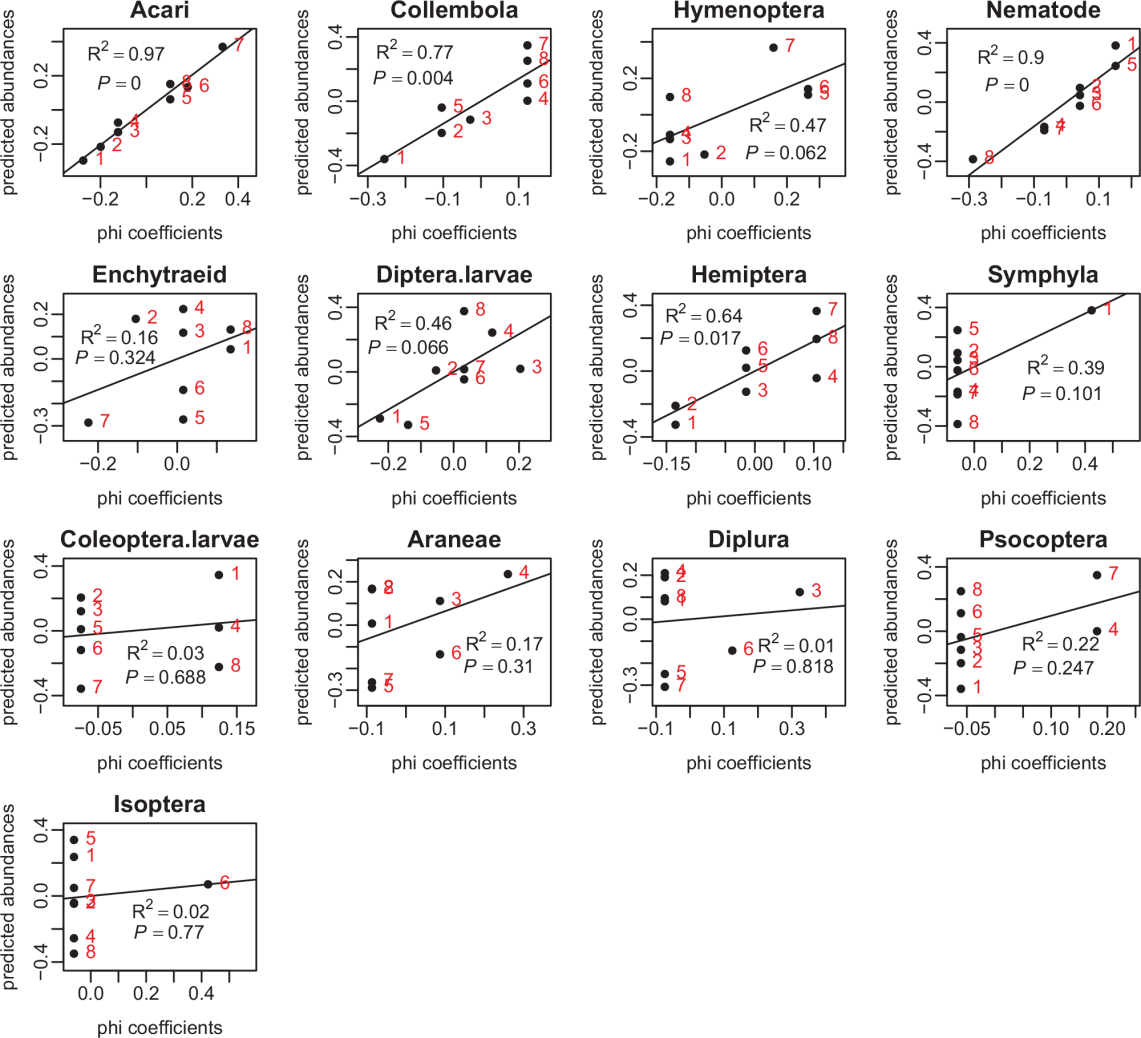
Relationships between predicted abundances and phi coefficients of soil fauna. Codes for habitats: 1 –ASa; 2 –ASb; 3 –BSa; 4 –BSb; 5 –CSc; 6 –CSd; 7 –DSc; 8 –DSd.

Through linear regression analyses, relationships between phi coefficients and predicted abundances of soil fauna were determined. Fig 5 shows that the predicted abundances were positively correlated with phi coefficients for Acari (R^2^ = 0.97, *P* = 0.000), Collembola (R^2^ = 0.77, *P* = 0.004), Nematode (R^2^ = 0.90, *P* = 0.000), Diptera larvae (R^2^ = 0.46, *P* = 0.066), and Hemiptera (R^2^ = 0.64, P = 0.017).

## Discussion

### Evaluating the applicability of phi coefficient in indicating habitat preference of soil fauna

Although ecologists have recommended phi coefficient in determining ecological preferences [21], the application of it in soil ecology has not been reported. Lack of knowledge about their applicability impedes their use in determining the associations between soil fauna and habitats, despite their advantages [20]. Results in the present study implies the applicability of phi coefficient in evaluating habitat preferences of soil fauna and preference-habitat factors relationships.

The phi coefficients matrix could, to a great extent, represent information on fauna-habitat relationships and inter-taxa relationships expressed by ordination methods. A highly significant relationship between the phi coefficients matrix and predicted abundances matrix was shown by CCorA (Fig 4). The ecological patterns of both fauna-habitat and inter-taxa relationships expressed respectively by the phi coefficients matrix and predicted abundances matrix were extremely similar. Additionally, highly positive correlations between phi coefficients and predicted abundances for Acari, Collembola, Nematode and Hemiptera were observed (Fig 5). A low phi coefficient value indicates a weak association of organisms with the target habitat. Accordingly, in an ordination biplot, this weak association will be shown as a small projection value of the habitat on the species arrow. However, one advantage of phi coefficient is that the statistical significance of organism-habitat associations can be assessed by permutation tests [21], which is not possible with ordination methods for organism-habitat relationships. In ordination biplots, the relationship between a taxon and a habitat can be found by projecting the centroid of the habitat at a right angle on the species arrow, but the statistical significance of the relationship cannot be ascertained, as with phi coefficient.

Regressing phi coefficients of an taxon upon explanatory variables can quantitatively assess the strength and statistical significance of the organism-habitat factor relationships. Additionally, this assessment of organism-habitat factor relationships can be more robust than that based on abundance datasets through ordination methods. The presence/absence datasets give a more robust estimation of organism-habitat relationships than abundances datasets as they are less affected by temporal fluctuations and observer bias [19]. In ordination biplots, we can directly characterize the relationships between organisms and explanatory variables, and between organisms themselves or explanatory variables themselves in a qualitative way. However, we may prefer to ascertain quantitative relationships between organisms and explanatory variables. To achieve this, firstly we have to obtain the predicted abundances of organisms across habitats at the expense of having to re-compute the projection values of centroids of habitats onto species arrows in the ordination biplots. Instead, conveniently with a phi coefficient approach, using the presence/absence dataset, quantitative relationships between habitat preferences and explanatory variables can be easily obtained, which to some extent, reflect the relationships between organisms and explanatory variables. In the present study, soil fauna showed complicated requirements for habitat conditions (Fig 3). The phi coefficient-habitat factor relationships showed that both habitat preferences of Acari and Hymenoptera were significantly dependent on TOC, N and C/N, and the habitat preference of Acari showed more dependence on these factors. In addition, the habitat preferences of Acari and Collembola showed similar dependence on pH. Meanwhile, the habitat preferences of Collembola and Nematode showed divergent dependence on DOC. Consistent with the present study, previous studies reported analogous requirements of Acari for higher soil TOC and N [10] and lower pH [8, 11], requirements of Collembola for higher TOC [37] and lower pH [11].

For some fauna taxa, in ordination biplots, their relationships with habitats and habitat factors will be overlooked due to their positions clustering around the origin with shorter species arrows. In a phi coefficient approach, however, these relationships may be easily observed. In the present study, the position of Hemiptera in the ordination biplot was near the origin of the coordinates, and nothing could be expressed about its relationship with habitats, soil chemical variables or other taxa. However, linear regression analysis showed a significantly negative correlation between its phi coefficients with soil pH (Fig 3), which would not be the case in ordination plots (Fig 2).

Habitat preferences characterized by phi coefficients could reflect the distribution states of soil fauna. The computation of a phi coefficient value is based on presence-absence data and the formula includes two important parameters, i.e., *n*_*p*_ and *N*_*p*_, where *n*_*p*_ is the number of occurrences of the species within the target site group and *N*_*p*_ is the number of sites belonging to the target site group (Table 1). The phi coefficient is directly determined by the frequency of occurrences within groups of sites provided that *N*_*p*_ is equivalent across site groups. This relationship can be shown by the scatter plots of phi coefficients against frequency (S1 Fig), implying that phi coefficients can reflect the distribution states of soil fauna. The dependence of phi coefficients on *n*_*p*_ and *N*_*p*_ induces inconsistency between the ordering of some habitats produced by phi coefficients and that produced by the predicted abundances. For instance, the phi coefficients of Collembola associated with habitats BSb, CSd, DSc, and DSd were equivalent to each other, while the predicted abundances within these habitats had differences, i.e., DSc > DSd > CSd > BSb (Fig 5). The same values of phi coefficients for Collembola with habitats resulted from the equivalent values of *n*_*p*_ and *N*_*p*_ among these habitats. The previous studies usually plotted incidence against abundance-when-present (omitting samples with zero individuals from the calculation of mean abundance) and observed significantly positive correlations between abundance and incidence [38]. In this way, the correlations between abundance and incidence for rare taxa would also be significant. Nevertheless, in the present study, the absences of taxa were also taken into consideration in partial RDA and the calculation of phi coefficients, which may be why the strong relationships between phi coefficients and predicted abundances were mostly confined to abundant taxa instead of rare taxa (Fig 5).

### The potentially extensive application of phi coefficient

Researchers have found that when applying ordination methods to abundance and presence–absence data independently, a more complete understanding of the ecological patterns was obtained and both data formats could be complementary [39]. At different levels of numerical resolution, such as absolute abundance, abundance rankings, and the presence and absence data, different viewpoints of the organism-habitat relationships will be obtained[40]. This can bring about a more comprehensive understanding of the driving factors in the composition of organism assemblages and biodistributions. The present study also highlights the importance of spontaneously utilizing abundance and presence–absence data to frame comprehensive ecological patterns of organism-habitat (factor) relationships. Here we suggest that, like studying biodiversity which usually consists of richness and evenness components [41], when considering organism-habitat relationships, not only the ordering of habitats on the abundance of organisms but also their distribution should be considered. Finally, the phi coefficient-induced results, such as ecological preferences and the associated quantitative relationships with habitat factors, may be largely complementary to the results of ordination methods.

The correlations between phi coefficients and predicted abundances can be an alternative approach for the study of relationships between the distribution and abundance of species. The reason is that both phi coefficients and predicted abundances, to some extent, involve general features of the ecological patterns rather than the previous simple calculation of incidence and mean abundances of species. Researchers have noted the limited utility of simple correlations between distribution and abundance [38]. The phi coefficient takes into account absences of the species outside the target site group and is dependent on the relative size of the target site group. With respect to predicted abundance of a given species, the calculation is based on its position in the ordination biplot. The position of the species in a biplot is an integrated consequence of its relationships with other species and explanatory variables as well as habitats.

### The necessity for further evaluating the applicability of phi coefficient

The phi coefficient was originally used to indicate the degree of association of organism assemblages with site groups that can represent habitats instead of simply indicating the existence of organisms or not at a species level [19, 21]. However, in the present study, the application of phi coefficient for habitat preference of soil fauna was evaluated at coarse levels of taxonomic resolution. The results implies that, using coarse taxonomic data, phi coefficient may be applicable in indicating the strength of habitat preferences based on which quantification of the relationships between preferences and habitat factors can be conducted. The coarse level of taxonomic resolution is indispensable and appropriable to some extent, because the large abundance and great phylogenetic diversity of soil fauna, and thus the identification of soil fauna to fine taxonomic resolution, such as at a species level, can be a difficult and laborious task [42]. While fine taxonomic resolution could provide higher specificity for the relationships of organisms with habitats and habitat conditions at the cost of a laborious task of identification, data of coarse taxonomic resolution can be appropriate in practice. Coarse taxonomic resolution has been considered an alternative proposed to reduce time and economic efforts [43, 44] and can provide valuable investigation of the effects of environmental factors and management measures. However, the applicability of phi coefficient imperatively not only should be evaluated at coarse levels of taxonomic resolution, like in the present study, but should also be evaluated in detail regarding taxonomic resolutions and functional groups, such as varying species and feeding types. A view based on phi coefficient in more detail on different soil fauna taxa will probably gain new and interesting insights about their habitat preferences and the related relationships with habitat factors. Additionally, in the present study, the evaluation of phi coefficient was conducted only based on a single field experiment, so the applicability of phi coefficient in other places and scales apart from our local site should be further evaluated.

## Conclusions

Through comparing phi coefficient-induced results with those generated from the constraint ordination method about relationships between soil fauna and habitat(factors), we suggest that phi coefficient could be applicable on a local scale in quantifying the degree of habitat preferences of soil fauna at coarse taxonomic levels.Based on presence-absence data, phi coefficient-induced results, e.g. the degree of habitat preferences and the associated quantitative relationships with habitat factors may be largely complementary to the outputs of ordination methods. Additionally, the correlations between phi coefficients and predicted abundances from ordinations will probably provide a new and useful viewpoint for the study of distribution-abundance relationships. The application of phi coefficients in soil ecology will benefit the understanding of biodistributions and variations in soil community composition, which may present a lot of interest for conservation biology, forest management and other applications. However, further evaluation of the application of phi coefficient in other places and scales, at fine taxonomic levels or in detail regarding functional groups, should be conducted and will probably obtain meaningful insights about the associations of soil fauna with habitat (factors).

## Supporting Information

S1 Fig Relationships between phi coefficients and frequencies of occurrenceshowed by the scatter plots. Codes for habitats: 1 –ASa; 2 –ASb; 3 –BSa; 4 –BSb; 5 –CSc; 6 –CSd; 7 –DSc; 8 –DSd. The phi coefficient will be directly determined by the frequency of occurrences within groups of sites, provided that site groupshave the same sizes. And this relationship can be showed by the scatter plots of phi coefficients against frequency, which implies phi coefficient can reflect the distribution states of soil fauna.
(EPS)

S1 Source Data The original data used in this study. (XLSX)

S1 Table The differences in abundances (A, ind. m^-2^) and relative abundances (RA, %) of main soil fauna taxa between habitats. Data are showed as mean ± SEM. Means with different letters indicate significant differences at α = 0.05 based on Kruskal-Wallis test.
(XLSX)
